# Clinical and arthroscopic findings in recreationally active patients

**DOI:** 10.1186/1758-2555-2-2

**Published:** 2010-01-15

**Authors:** Elizabeth M Fowler, Ian G Horsley, Christer G Rolf

**Affiliations:** 1 Centre for Health, Sport and Rehabilitation Sciences Research, University of Salford, Fredrick Road, Salford, UK; 2 Sheffield Centre of Sports Medicine, School of Biomedical Sciences, The University of Sheffield, Sheffield, UK

## Abstract

**Objective:**

To examine the diagnostic accuracy of standard clinical tests for the shoulder in recreational athletes with activity related pain.

**Design:**

Cohort study with index test of clinical examination and reference standard of arthroscopy.

**Setting:**

Sports Medicine clinic in Sheffield, U.K.

**Participants:**

101 recreational athletes (82 male, 19 female; mean age 40.8 ± 14.6 years) over a six year period.

**Interventions:**

Bilateral evaluation of movements of the shoulder followed by standardized shoulder tests, formulation of clinical diagnosis and shoulder arthroscopy conducted by the same surgeon.

**Main Outcome Measurements:**

Sensitivity, specificity, likelihood ratio for a positive test and over-all accuracy of clinical examination was examined retrospectively and compared with arthroscopy.

**Results:**

Isolated pathology was rare, most patients (72%) having more than one injury recorded. O'Brien's clinical test had a mediocre sensitivity (64%) and over-all accuracy (54%) for diagnosing SLAP lesions. Hawkins test and Jobe's test had the highest but still not impressive over-all accuracy (67%) and sensitivity (67%) for rotator cuff pathology respectively. External and internal impingement tests showed similar levels of accuracy. When a positive test was observed in one of a combination of shoulder tests used for diagnosing SLAP lesions or rotator cuff disease, sensitivity increased substantially whilst specificity decreased.

**Conclusions:**

The diagnostic accuracy of isolated standard shoulder tests in recreational athletes was over-all very poor, potentially due to the majority of athletes (71%) having concomitant shoulder injuries. Most likely, this means that many of these injuries are missed in general practice and treatment is therefore delayed. Clinical examination of the shoulder should involve a combination of clinical tests in order to identify likely intra articular pathology which may warrant referral to specialist for surgery.

## Background

Athletic injuries to the shoulder are both common and unique, being both difficult to diagnose and manage [1]. The majority of shoulder injuries occur from direct or indirect trauma or as a result of repetitive use [1] with the most frequently presenting shoulder conditions at primary care being rotator cuff pathology, glenohumeral and acromioclavicular joint disorders and pain referral from the neck [2]. The most commonly reported shoulder pathology in older athletes is rotator cuff pathology [1,2] with 85% of patients at primary care level being diagnosed with this pathology [3]. Obtaining a specific diagnosis, in addition to determining the prognosis and appropriate treatment strategy for a patient is essential [4] particularly considering the resolution of shoulder conditions is extremely poor with 41% of primary care patients having persistent pain 12 months post initial consultation [5]. Fundamental to shoulder disorder diagnosis is the lack of accepted diagnostic criteria [4]. Whilst numerous clinical examination tests for the shoulder exists, some of which were specifically designed to identifying specific disorders, such as O'Brien's test for labral lesions [6], difficulty exists in diagnosing and differentiating shoulder disorders by one single test [7].

Whilst the diagnostic accuracy of various clinical tests in identifying specific shoulder pathologies has been widely discussed in previous research [8-12], some of the research only selected and analysed patients with a specific condition in mind [10]. Consequently the probability of a test diagnosing a patient who has the pathology is extremely high, resulting in the test being assessed in an already biased setting. Whilst this ensures the clinical tests assessed are specific to identifying a particular pathology when that is known, it does not necessarily mean that the test is accurate to demonstrate this injury in a clinical setting when people clinically present with undiagnosed shoulder pain. For the non specialist clinician, the main aim of such clinical examination, utilizing specific shoulder tests, is to try to establish a working diagnosis and thereby distinguish between cases requiring surgical referrals and cases where conservative treatment may be sufficient. It is obvious that the more structures that are injured in the shoulder the more problematic this examination will be to interpret. This article draws attention to the fact that upon patent presentation it is difficult to surmise how many patients will present with "isolated and specific injuries" or have more complex scenarios.

To our knowledge no study has examined clinical tests for the shoulder undertaken on consecutive recreational athletes within a routine clinical setting, without any exclusion criteria. The definition of a recreationally active patient for inclusion in this study was; a person who took part in competitive sport, of a non-professional nature, on three or more occasions a week. The aim of this paper was to assess the diagnostic accuracy of routine clinical tests for the shoulder in consecutive recreationally active patients, presenting in a sports medicine clinic.

## Methods

A total of 101 recreationally active patients were examined by a senior orthopaedic consultant and after examination underwent arthroscopic surgery between January 2001 and December 2006. All data was subsequently entered into a standardized database and retrospectively analysed as a consecutive subjects design. Prior to assessment, consent was obtained in writing from each patient and ethical approval was obtained from the University of Sheffield, U.K. The same orthopaedic surgeon conducted all physical examinations and arthroscopies.

Bilateral evaluation of all active, passive and resisted movements of the shoulder was a pre-requisite to the physical assessment. A battery of routine shoulder tests were incorporated into the examination in all patients; these were O'Brien's test [11], Jobe's test [13], Hawkins-Kennedy test [14], Palm-up test [15], Apprehension-relocation test [6] and Gerber's lift-off test [16].

Following subjective and physical examination findings, a clinical diagnosis was formulated. Where deemed appropriate, further investigation was prescribed involving either an x-ray or MRI scan. An arthroscopy was undertaken later by the same consultant, whereby all procedures were recorded onto DVD. The operative findings were routinely documented, citing all intra articular structural pathology and outcomes of functional intra-operative dynamic assessments referring to internal and external impingement. Shoulder arthroscopy was undertaken as a day case, with no complications reported in any patient. All patients were referred post-operatively for physiotherapy, within the orthopaedic surgeon's supportive team.

All patient documentation from initial consultation to subsequent discharge, was systematically audited and analysed, retrospectively, by an unbiased observer, uninvolved in any stage of the treatment given to patients. The data was analysed using the software, Statistical Package for the Social Sciences (SPSS; version 15.0) where assessment of diagnostic test accuracy for all tests in various pathologies was calculated using a 2 × 2 table. Test sensitivity, specificity, positive predictive value (PPV), negative predictive value (NPV), likelihood ratio for a positive test and overall accuracy were calculated [17].

## Results

There was 101 recreational athletes with shoulder pathology (82 male, 19 female), mean age 40.8 (+/- 14.6 years) with 58% (n = 59) presenting with right shoulder pain. The patients, who were all keen participants in sport, competing in their chosen activity three or more times per week in addition to being employed full-time, were regarded as being recreationally active. A total of 92% of the cohort complained of activity-related pain whilst pain on overhead activities was reported in 89% (n = 90) of patients.

The average time from injury to initial orthopaedic consultation was 35.6 weeks with 75% of patients attending physiotherapy prior to the assessment. All 101 recreational athletes underwent arthroscopy, waiting an average of 3.6 weeks from orthopaedic consultation to surgery. Of the entire recreational cohort, 71% (n = 72) of patients had more than one shoulder pathology present, as verified by arthroscopy. The most common operative finding was a grade II or III SLAP lesion, evident in 50.4% (n = 51) of patients with only 7 patients having an isolated SLAP lesion. Rotator cuff pathology, defined as partial under surface tears or complete tears, with or without macroscopic degenerative changes, was detected in a total number of 35 patients (35%) with only three patients presenting an isolated rotator cuff injury. Thirty-one cases (30%) of internal impingement (characterised by pinching of flap tears from the SLAP and/or under surface rotator cuff and/or subscapularis between the humeral head and the glenoid on passive overhead simulation-patients in beach chair position) were reported with none occurring in isolation. External impingement (characterised by compression of the rotator cuff tendons against the acromion on passive abduction and internal rotation) was present in 28% (n = 28) of patients and was the most common operative finding occurring in isolation with 11 reports of this pathology. No normal arthroscopies were reported.

### Diagnostic Accuracy of Clinical Tests

The diagnostic accuracy of clinical examination tests for the shoulder, as verified by arthroscopy, using sensitivity, specificity, positive predictive value (PPV), negative predictive value (NPV), likelihood ratio for a positive test and over-all accuracy are presented in Table 1.

**Table 1:** Over-all diagnostic values of clinical tests for shoulder pathology in recreational athletes.

Arthroscopy finding	Clinical preoperative test	Sensitivity (%)	Specificity (%)	PPV (%)	NPV (%)	Likelihood Ratio	Over-all Accuracy (%)
SLAP lesion	Hawkins	30.0	53.3	41.7	40.7	0.64	41.1

	Jobe's	60.0	35.6	50.8	44.4	0.93	48.4

	Palm up	47.4	55.6	47.4	55.6	1.06	51.8

	O'Brien's	64.0	43.2	56.1	51.4	1.13	54.3

	Apprehension	28.6	68.9	50.0	47.0	0.92	47.9

	Gerber's	12.2	86.7	50.0	47.6	0.92	47.9

Rotator cuff pathology	Hawkins	57.6	72.1	52.8	75.9	2.06	67.0

	Jobe's	66.7	41.0	37.9	69.4	1.13	50.0

	Palm up	45.5	62.3	39.5	67.9	1.21	56.4

	O'Brien's	54.5	35.0	31.6	58.3	0.84	41.9

	Apprehension	21.9	62.5	22.6	61.5	0.58	49.0

	Gerber's	18.8	90.2	50.0	67.9	1.91	65.6

Labrum tear	Hawkins	26.3	59.2	13.9	76.3	0.65	52.6

	Jobe's	57.9	36.8	18.6	77.8	0.92	41.1

	Palm up	63.2	65.8	31.6	87.7	1.85	65.3

	O'Brien's	63.2	40.0	21.1	81.1	1.05	44.7

	Apprehension	31.6	70.7	21.4	80.3	1.07	62.8

	Gerber's	20.0	88.9	50.0	66.7	1.80	64.3

Bankart lesion	Apprehension	79.2	87.1	67.9	92.4	6.16	85.1

Hill Sachs	Apprehension	81.3	80.8	46.4	95.5	4.23	80.9

NPV = Negative predictive value.

PPV = Positive predictive value.

### SLAP lesions

The sensitivity and specificity of tests utilised to diagnose SLAP lesions, within this study, ranged from 12% to 64% and 36% to 87% respectively (Table 1). O'Brien's test had mediocre sensitivity (64%), PPV (56.1%) and over-all accuracy (54.3%).

Gerber's lift off test reported highest specificity (86.7%), whilst Palm-up test had highest NPV (55.6%) for SLAP lesions. The highest likelihood ratio for a positive test in patients with a SLAP lesion was 1.13 for O'Brien's test (Table 1).

When a positive response was observed in either Jobe's, O'Brien's or Palm-up test, sensitivity for detecting SLAP lesions increased to 94%, whilst specificity subsequently plummeted to 18% (Table 2). Specificity on the whole, when combinations of tests were utilised, where a positive response in one test was reported, decreased to between 18% and 33%; results very dissimilar to when these tests were analysed in isolation (range 36%-87%). The highest over-all accuracy reported when a positive response was observed in one of Jobe's, Palm-up and O'Brien's tests was 58%; results not too dissimilar to that of O'Brien's test utilised in isolation (54%). NPV and PPV slightly increased over-all when a positive reporting in one test of a combination was analysed.

**Table 2:** Diagnostic accuracy when one of the combinations of shoulder clinical examination tests utilised to diagnose SLAP lesions as verified by arthroscopy, is positive during clinical examination, in recreational athletes.

Clinical tests	Sensitivity (%)	Specificity (%)	PPV (%)	NPV (%)	Likelihood ratio	Over-all accuracy (%)
Jobe's + Palm-up + O'Brien's	94.00	17.78	55.95	72.73	1.14	57.89

Jobe's + O'Brien's	91.84	17.78	54.88	66.67	1.12	56.38

Palm-up + O'Brien's	76.00	30.43	54.29	53.85	1.09	54.17

Palm-up + Jobe's	70.59	32.65	52.17	51.61	1.05	52.00

### Rotator Cuff Pathology

Rotator cuff pathology, as verified by arthroscopy, was not diagnosed accurately over-all by Hawkins test (67%) (Table 1), whilst the likelihood ratio was the highest of all tests at 2.06. This clinical test also showed a PPV of 53%, NPV of 76% and sensitivity of 58%; the latter of which was preceded slightly by Jobe's test (67%). Specificity ranged from 35% to 90% for the shoulder tests, the latter of whish corresponded to Gerber's lift-off test.

Test sensitivity for diagnosing rotator cuff pathology, increased to 97% when a combination of Hawkins, Jobe's or O'Brien's test was positive during clinical examination; a result corresponding to very low specificity (10%), with Hawkins and Palm-up test attaining a specificity value of 49% (Table 3). A positive response in one of Hawkins, Jobe's or Palm-up tests resulted in a high NPV (89%), whilst Hawkins and Palm-up had over-all accuracy for diagnosing rotator cuff pathology of 46% and 60% respectively.

**Table 3:** Diagnostic accuracy when one of the combinations of shoulder clinical examination tests utilised to diagnose rotator cuff pathology, as verified by arthroscopy, is positive during clinical examination, in recreational athletes.

Clinical Tests	Sensitivity (%)	Specificity (%)	PPV (%)	NPV (%)	Likelihood ratio	Over-all accuracy (%)
Hawkins + Jobe's + O'Brien's	96.88	9.84	36.05	85.71	1.07	39.78

Hawkins + Jobe's + Palm-up	93.94	27.87	41.33	89.47	1.30	51.06

Hawkins + O'Brien's + Palm-up	90.91	16.39	37.04	76.92	1.09	42.55

Jobe's + Palm-up + Obrien's	90.91	13.11	36.14	72.73	1.03	40.43

Hawkins + O'Brien's	87.50	19.67	36.36	75.00	1.09	43.01

Jobe's + O'Brien's	87.50	13.11	34.57	66.67	1.01	38.71

Hawkins + Jobe's	81.82	33.87	39.71	77.78	1.24	50.53

Palm-up + Jobe's	80.00	37.50	41.18	77.42	1.28	52.53

Hawkins + Palm-up	79.41	49.21	45.76	81.58	1.56	59.79

Palm-up + Obrien's	75.76	27.42	35.71	68.00	1.04	44.21

### Labral tear

The most sensitive tests for identifying a labrum tear in general (including SLAP) were both O'Brien's test and Palm-up test (63%), with the latter reporting an over-all accuracy 65.3% and NPV 87.7%, in addition to having the highest likelihood ration (1.85) (Table 1). Gerber's lift off test had the highest specificity (88.9%). When a positive clinical finding was observed in Jobe's, Palm-up an d O'Brien's test used in combination, test sensitivity increased to 94.7% (Table 4). However, an identical result was obtained when a positive response in either Jobe's test or Palm-up test was reported (94.7%). Specificity subsequently decreased with the highest value recorded as 33% using Palm-up and Jobe's tests. PPV and NPV on the whole, increased, whilst over-all accuracy for diagnosing labral tears was found to decrease to values between 29% and 42%.

**Table 4:** Diagnostic accuracy when one of the combinations of shoulder clinical examination tests utilised to diagnose labral tears, as verified by arthroscopy, is positive during clinical examination, in recreational athletes.

Clinical Tests	Sensitivity (%)	Specificity (%)	PPV (%)	NPV (%)	Likelihood ratio	Over-all accuracy (%)
Jobe's + O'Brien's	94.74	14.67	21.95	91.67	1.11	30.85

Jobes + Palm-up + O'Brien's	94.74	13.16	21.43	90.91	1.09	29.47

Palm-up + O'Brien's	84.21	29.87	22.86	88.46	1.20	40.63

Palm-up + Jobes	78.95	33.33	21.74	87.10	1.18	42.00

### Internal Impingement

The over-all diagnostic accuracy of tests to determine the presence of internal impingement in recreational athletes ranged from 46% to 64%, with Gerber's test attaining the highest test accuracy (Table 5). Hawkins test however, cited the highest specificity and NPV (66% and 73% respectively) with Jobe's test being the most sensitive (63%).

**Table 5:** Over-all diagnostic values of clinical tests for internal and external impingement in recreational athletes.

Arthroscopy finding	Clinical preoperative test	Sensitivity (%)	Specificity (%)	PPV (%)	NPV (%)	Likelihood ratio	Over-all accuracy (%)
Internal impingement	Hawkins	46.7	66.2	38.9	72.9	1.38	60.0

	Jobe's	63.3	38.5	32.2	69.4	1.03	46.3

	Palm up	46.7	63.1	36.8	71.9	1.26	57.9

	O'Brien's	63.3	40.6	33.3	70.3	1.07	47.9

	Apprehension	13.3	62.5	14.3	60.6	0.36	46.8

	Gerber's	13.3	87.5	33.3	68.3	1.07	63.8

External impingement	Hawkins	60.0	70.0	41.7	83.1	2.0	67.4

	Jobe's	76.0	42.9	32.2	83.3	1.33	51.6

	Palm up	48.0	62.9	31.6	77.2	1.29	58.9

	O'Brien's	36.0	30.4	15.8	56.8	0.52	31.9

	Apprehension	12.5	64.3	10.7	68.2	0.35	51.1

	Gerber's	12.5	87.1	25.0	74.4	0.97	68.1

NPV = Negative predictive value.

PPV = Positive predictive value.

### External Impingement

Jobe's test was the most sensitive test, with the highest NPV at diagnosing external impingement in a pathological shoulder (76% and 83% respectively), being 16% more sensitive than Hawkins test; the latter of which had the highest specificity, PPV and likelihood ratio (70%, 42% and 2.0 respectively) (Table 5).

### Anterior Instability

In the entire recreational cohort, the Apprehension test had high sensitivity and specificity values for diagnosing Bankart lesions (79% and 87% respectively) and Hill Sachs lesions (81.5% and 80.7% respectively), in addition to having high likelihood ratios for both conditions (6.16 and 4.23 respectively) (Table 1).

## Discussion

Clinical examination tests aim to screen, diagnose, grade and monitor the progression of a disease [18]. The accuracy of numerous clinical tests for the shoulder with respect to arthroscopic findings is extremely variable in both this study and previous literature [8-10,12]. The primary focus of this paper was to determine the diagnostic accuracy of standard clinical tests for shoulder pathology in consecutive recreationally active patients with shoulder pain in relation to arthroscopic findings. This patient category is common in most general practices around the UK and their injuries are, in our experience, often missed and not referred for orthopaedic specialist evaluation. The fact that most patients suffered from more than one pathology may blur the perception and clinical diagnosis, which may lead to wrong conclusions for further treatment; in particular requirements for surgery.

A SLAP lesion is a relatively unique condition, with disagreement amongst authors as to the most provocative test to identify this pathology [10-12]. Whilst numerous authors have previously discussed the diagnostic accuracy of O'Brien's clinical test for the shoulder [10-12], it must be noted that the substantial diagnostic accuracy reported by O'Brien et al [11] for this test was based on its ability to assess labral abnormalities. Parentis et al [12] analysed O'Brien's test specific to SLAP lesions and reported adequate sensitivity (62.5%) as did this current study (64%). An observation of O'Brien's test being more sensitive than specific is also evident between this investigation and the aforementioned study, a finding contradictory to the findings of McFarland et al[10] (sensitivity 47%, specificity 55%). However, McFarland et al[10] had the same over-all accuracy value for O'Brien's test diagnosing SLAP lesions (54%) as that reported here. Whilst this study cited findings similar to previous research for assessing O'Brien's accuracy in diagnosing SLAP lesions, several discrepancies exist between these studies.

McFarland et al[10] reported the diagnostic tests for SLAP lesion identification (compression/rotation test, O'Brien's and anterior slide test) were positive at almost the same rate in the control and SLAP lesion groups utilised in their study, consequently querying the validity of O'Brien's test as a diagnostic aid for this pathology. However, McFarland et al[10] allocated patients with Type I SLAP lesions into the control group of the study, as the authors regarded this condition as being degenerative and un-related to mechanical symptoms. Considering Kim et al[19] reported 74% of 139 patients suffering from SLAP lesions were Type I, McFarland et al[10] appear to have excluded a substantial proportion of the population, although neither study restricted their subject population to recreational athletes. Parentis et al[12] utilised a less extreme exclusion criteria omitting patients with adhesive capsulitis and those who were unable to complete a physical examination. This investigation however, analysed all patients irrespective of severity and type of shoulder pathology, including all individuals with SLAP lesions.

Despite O'Brien's test having on average, the highest sensitivity, likelihood ratio, PPV and over-all accuracy for diagnosing SLAP lesions in this study, these values are mediocre and consequently this study is unable to validate this test, or any other, as a single accurate, reliable tool for diagnosing SLAP lesions. Considering 50% of patients had SLAP lesions in this study, with 86% having concomitant injuries with the SLAP lesion, it is feasible that it was going to be unachievable to diagnose SLAP lesions utilising only O'Brien's test. No one test utilised in the clinical examination in this study, attained high values in all diagnostic categories thereby suggesting that no single test is both sensitive and specific in diagnosing SLAP lesions, a conclusion supported by Parentis et al [12]. Oh et al [20] recommended combining two relatively sensitive clinical tests and one relatively specific clinical test as a means of increasing the diagnostic accuracy for detecting SLAP lesions.

With Hawkins clinical test being considered as one of the most commonly used tests to assess for external shoulder impingement [9], of which rotator cuff pathology is a potential contributor to this condition [21], this test is subsequently discussed in relation to rotator cuff disease. Clinical tests utilised to diagnose rotator cuff pathology are generally more sensitive than specific [8] a fact evident in the findings of MacDonald et al [9] whereby sensitivity exceeded specificity by 45% for Hawkins test, whilst Bin Park et al [8] reported a similar trend (sensitivity 72%, specificity 66%). This particular investigation had contradictory trends with specificity being 15% higher than sensitivity. Remarkably similar over-all accuracy results for Hawkins test and rotator cuff disease are evident however between this study and that of Bin Park et al [8] (67% and 69.7% respectively), and a likelihood ratio of 1.85. However, Bin Park et al [8] utilised a patient population that had the diagnosis of subacromial syndrome; a condition defined in the study as patients with partial and full rotator cuff tears, bursitis, or rotator cuff tendonitis. Patients suffering from impingement with concomitant injuries such as SLAP lesions or instability were excluded. Whilst this ensures the diagnostic accuracy of shoulder tests are specific to rotator cuff pathology, or bursitis, it nonetheless excludes a vast majority of patients from a study. Murrell and Walton [22] reported several clinical tests for the shoulder as being unable to distinguish between patients with rotator cuff tears and alternate shoulder pathologies. Over-all, this study cannot promote the use of one single clinical test to specifically diagnose rotator cuff pathology.

A result to possibly note is the high specificity and NPV associated between Hawkins test and internal impingement (66% and 73% respectively). Considering specificity and NPV relate to a test being able to predict the absence of pathology in patients without the disease, it is unsurprising Hawkins attained high scores in these categories. However, as Pappas et al [23] reported that internal impingement can be elicited by both Neers and Hawkins tests, it may explain why the latter test obtained 60% over-all accuracy for internal impingement of the shoulder. The role of Hawkins test in diagnosing internal impingement is unclear and warrants further investigation. It may simply be due to the proximity between the SLAP and under surface of the rotator cuff compressing an impinging flap during the test. This finding may also be of importance in respect to the relatively common situation whereby a suspected subacromial impingement is treated arthroscopically but not successfully, due to a missed SLAP tear impingement in the glenohumeral joint.

Although a number of clinical tests used for the diagnosis of painful shoulder are considered accurate in determining the location of the periarticular lesions, these entities may be difficult to differentiate by physical examination [24]. The inability of isolated clinical tests for the shoulder, to assess for specific shoulder disorders is evident in this study, with no one test being significantly outstanding for detecting any condition. This study further demonstrated that a positive response gained in one of a combination of clinical tests caused test sensitivity to increase substantially in all pathological conditions, with specificity subsequently plummeting.

A positive response in one or more of Jobe's, O'Brien's or Palm-up tests, when assessed together, was the most sensitive for diagnosing intra articular pathology such as both SLAP lesions (94%) and labral tears (95%). Jobe's and O'Brien's test elicited the same sensitivity result when diagnosing labral tears (95%) in addition to being only 2% lower in sensitivity for SLAP lesions. Whilst sensitivity values are extremely impressive when the clinician reports one or more of a battery of tests to be positive for various pathologies, specificity is subsequently extremely poor with Jobe's and O'Brien's tests being only 45% and 18% specific for labral and SLAP lesions respectively. Even though the exact diagnosis is vague, surely a finding like this would render a referral to orthopaedic surgeon with the query of arthroscopic intervention.

Diagnosing rotator cuff pathology using a positive response in one or more of either Jobe's, O'Brien's or Palm-up tests resulted in sensitivity of 91%. The substitution of O'Brien's test with Hawkins clinical test resulted in a 3% and 11% increase in sensitivity and over-all accuracy respectively. The high sensitivity values were accompanied by extremely poor specificity results. With minimal differences observed between the aforementioned results for sensitivity, this highlights not only the ability of these tests in detecting the presence of shoulder pathology, but more importantly, emphasises their ineffectiveness at differentiating different shoulder pathologies.

The majority of previous research analysing the diagnostic accuracy of clinical tests for the shoulder, implemented varying degrees of exclusion criteria for patients. Previous research eliminated patients with concomitant shoulder injuries [8,10] which is potentially unwise considering the wide spectrum of shoulder disease which may present in a patient, as evident in this study. Having a large variation in subject pathology in this study gives a good over-view as to how the clinical tests for the shoulder may perform in other professional settings [25].

Despite clinical tests being an integral part of the over-all examination procedure [26] utilising these tests in isolation or combination, in recreational athletes, must be cautioned, considering the poor diagnostic accuracy presented in this study. Considering that clinical diagnostic tests for the shoulder are most often undertaken in general practice and can be positive in the presence of other conditions [8], as evident in this investigation, the clinician should consider the results of the examination on the basis of the clinical presentation and subjective history of the patient. It is recommended that a systematic evaluation, including a combination of tests be utilised to establish an accurate working diagnosis for shoulder injuries [7] of which positive findings within these tests may warrant further investigations by a specialist.

## Limitations

The present study involved a retrospective review of the information collected by one surgeon. Although the surgeon did not have access to the results of the clinical tests at the time of the surgery, lack of independence of clinical tests and surgical findings, introduces a potential bias related to blindness. This study involved only surgical patients. Patients with potential pathology who had negative tests and did not have surgery were not included in analysis. This may indicate a verification or work-up bias, causing inflation of sensitivity and underestimating specificity.

## Competing interests

The authors declare that they have no competing interests.

## Authors' contributions

CR participated in the design and co-ordination of the study and conducted the physical examination of all patients in addition to undertaking the arthroscopic surgery in the entire population. EF entered the findings of the physical examination and arthroscopy into a standardized database and undertook the statistical analysis. IH participated in the design, co-ordination and analysis of the study and the drafting and revising of the manuscript. All authors read and approved the final manuscript.
